# The crucial role of lateral root angle in enhancing drought resilience in cotton

**DOI:** 10.3389/fpls.2024.1358163

**Published:** 2024-02-05

**Authors:** Congcong Guo, Xiaoyuan Bao, Hongchun Sun, Jing Chen, Lingxiao Zhu, Jianhong Zhang, Haina Zhang, Yongjiang Zhang, Ke Zhang, Zhiying Bai, Anchang Li, Liantao Liu, Cundong Li

**Affiliations:** ^1^ State Key Laboratory of North China Crop Improvement and Regulation, Key Laboratory of Crop Growth Regulation of Hebei Province, College of Agronomy, Hebei Agricultural University, Baoding, Hebei, China; ^2^ Institute of Cotton Research of Chinese Academy of Agricultural Sciences, National Key Laboratory of Cotton Biology, Anyang, Henan, China; ^3^ Cotton Research Institute, Hebei Academy of Agriculture and Forestry Sciences, Shijiazhuang, China

**Keywords:** drought, lateral root angle, stomata, canopy temperature, cotton

## Abstract

**Introduction:**

Plant responses to drought stress are influenced by various factors, including the lateral root angle (LRA), stomatal regulation, canopy temperature, transpiration rate and yield. However, there is a lack of research that quantifies their interactions, especially among different cotton varieties.

**Methods:**

This experiment included two water treatments: well-watered (75 ± 5% soil relative water content) and drought stress (50 ± 5% soil relative water content) starting from the three-leaf growth stage.

**Results:**

The results revealed that different LRA varieties show genetic variation under drought stress. Among them, varieties with smaller root angles show greater drought tolerance. Varieties with smaller LRAs had significantly increased stomatal opening by 15% to 43%, transpiration rate by 61.24% and 62.00%, aboveground biomass by 54% to 64%, and increased seed cotton yield by 76% to 79%, and decreased canopy temperature by 9% to 12% under drought stress compared to the larger LRAs. Varieties with smaller LRAs had less yield loss under drought stress, which may be due to enhanced access to deeper soil water, compensating for heightened stomatal opening and elevated transpiration rates. The increase in transpiration rate promotes heat dissipation from leaves, thereby reducing leaf temperature and protecting leaves from damage.

**Discussion:**

Demonstrating the advantages conferred by the development of a smaller LRA under drought stress conditions holds value in enhancing cotton’s resilience and promoting its sustainable adaptation to abiotic stressors.

## 1 Introduction

Drought stress has a detrimental effect on agriculture (Abbas et al., 2018). Approximately one-third of the world’s arable land is characterized by arid or semiarid conditions and limited water resources, which reduce the annual crop yields by over 50% (Tuberosa and Salvi, 2006; Toker et al., 2007). Cotton (*Gossypium hirsutum* L.), as a globally significant cash crop, has been severely affected by drought, making it crucial to explore the strategies of crops to adapt to drought stress to enhance agricultural productivity (Chen et al., 2013).

The root is a vital organ that actively senses the soil environment. It acts as a conduit between the aboveground and the soil and is pivotal in nutrient and water uptake. Therefore, the development of root systems directly influences the aboveground growth. In addition, under drought stress, there is a highly significant correlation between the root system and aboveground development (Lynch, 2007; Erice et al., 2010), and this is garnering increasing attention from breeders (Trumbore and Gaudinski, 2003; Epstein, 2004; Sugden et al., 2004; Collins et al., 2008). The spatial structure and distribution of roots is known as the root system architecture (RSA) (Lynch, 2007). It is a crucial indicator for evaluating crop drought tolerance (Lynch and Brown, 2001; Lynch, 2013) since it significantly influences the plant’s adaptation to drought.

The RSA exhibits high plasticity and genetic diversity, which enables the root morphology to adapt to challenging environments (Rubio et al., 2003; Ochoa et al., 2006). An accurate description of the growth structure and spatial distribution of RSA in soil would allow an ideal RSA to more efficiently absorb water and nutrients from the soil environment and then distribute them to the plant aboveground part by signaling when resources are limited. For instance, research on wheat (*Triticum aestivum* L.) (MansChadi et al., 2010) and maize (*Zea mays* L.) (Lynch, 2013) has revealed that narrower/steeper root angles improve the root architecture, which leads to deeper roots and the enhanced uptake of deeper water resources. Furthermore, Uga et al. (2013) demonstrated that genotypes with narrower root angles and deeper root systems achieved higher seed yields under upland drought stress conditions than genotypes with shallower and wider angles. Roots exhibiting a steep growth angle potentially aid in accessing mobile nutrients, particularly nitrogen, present in deeper soil layers (Trachsel et al., 2013; Dathe et al., 2016; Liang et al., 2017). This growth pattern allows plants to tap into deep soil water, enabling them to sustain evapotranspiration needs during prolonged periods without additional water input. Thus, crops modulate their root system architecture, such as by reducing the lateral root angle (LRA) to more efficiently access water in the deeper soil environment, improve drought tolerance of the aboveground crop, and ultimately stabilize yields.

Stomatal regulation and leaf temperature influence the adaptation of plants under drought stress (Casson and Hetherington, 2010). Notably, the stomatal size and regulatory function along with the leaf photosynthesis and transpiration rates are significantly reduced under drought stress (Casson and Gray, 2008; Dong et al., 2018). Generally, the decrease in stomatal opening reduces the rate of plant transpiration and damage owing to drought stress (Xiong and Zhu, 2003). However, transpiration maintains stable leaf temperatures by lowering the leaf temperature (Testi et al., 2008). Therefore, decreased plant stomatal opening under drought stress increases the leaf temperature owing to the reduction in transpiration rate and increased water retention. Elevated leaf temperatures beyond the threshold disrupts the activity of protective enzymes, impairs the normal plant functions and ultimately leads to reduced crop yields (Fan et al., 2023). Therefore, changes in stomatal regulation and canopy temperature are often utilized as key indicators to monitor the stress levels of plants.

In summary, several factors of plants undergo significant changes, including LRA, stomatal regulation, canopy temperature, and yield under drought stress. However, the characteristics and interrelationships of these factors in cotton under drought stress are still understood. The hypothesis of the present study is that cotton varieties with smaller LRA experience reduced yield losses under drought stress, potentially due to enhanced water absorption from deeper soil layers, compensating for increased stomatal opening and transpiration rates. Specifically, the objectives of the study were (1) to explore the regulation of lateral root angle, (2) to elucidate the impacts of drought stress on stomatal traits, transpiration rates, and canopy temperature, and (3) to determine the response of LRA, stomatal traits, transpiration rates, canopy temperature, and yield under drought stress, while investigating their interrelationships.

## 2 Materials and methods

### 2.1 Plant materials

Eighty cotton varieties from diverse geographical regions were chosen as the study’s experimental materials in this study (**Supplementary Table 1**). The selection process took into account the geographical distribution and ecological adaptability of the varieties, with the objective of highlighting their broad distribution and rich diversity.

### 2.2 Experimental site

The experiment was conducted at Hebei Agricultural University’s Qingyuan Experimental Station in China (38.85°N, 115.30°E) during 2021 and 2022. Rain-out shelter were utilized in the field to minimize the impact of rainfall interference. The recorded average temperatures were 20.75°C with 2091.4 hours of sunshine in 2021, and 20.96°C with 2199.8 hours of sunshine in 2022 (**Supplementary Figure 1**). The soil is sandy loam with organic matter 13.83 g·kg^-1^, total nitrogen 0.93 g·kg^-1^, available nitrogen 69.45 mg·kg^-1^, available phosphorus 17.40 mg·kg^-1^ and available potassium 121.36 mg·kg^-1^. Details regarding soil bulk density and field water capacity are available in **Supplementary Table 2**.

### 2.3 Experimental design

This experiment included two water treatments: well-watered (WW), maintained at 75 ± 5% soil relative water content; and drought stress (DS), maintained at 50 ± 5% soil relative water content. The seeds were planted in a randomized complete block design with three replicates per treatment. The drought stress treatment was initiated at the three-leaf stage and continued until maturity. The irrigation method was micro-sprinkler. The volume of irrigation water was monitored using a water meter (with an accuracy of 0.0001 m^3^). During the study, under well-watered and drought stress treatments detectors were used soil water content, including Sensoterra (Soil moisture monitoring system, Netherlands) and TDR (Trime Pico 64 Portable Soil Moisture Meter, EMIKO, GmbH, Bochum, Germany). Sensoterra monitored the 0-30 cm soil water content (**Supplementary Figure 2**), and TDR monitored the 0-20 cm, 20-40 cm, and 40-60 cm soil water content over the entire growth period (**Supplementary Figure 3**).

### 2.4 Field management

Cotton was sown using the hole-sowing method on April 24, 2021, and April 24, 2022. The planting density was 90,000 plants hm^-2^ with a row spacing of 50 centimeters. Each plot received 450 kg ha^-1^ of compound fertilizer containing 15% nitrogen, 15% P_2_O_5_ and 15% K_2_O as base fertilizer. Additionally, 150 kg ha^-1^ of urea (containing 46% nitrogen) was top-dressed at the flowering stage. Other field management practices, aligned with the methods employed in local high-yield cotton fields.

### 2.5 Measurement methods

#### 2.5.1 Measurement of the plant height, aboveground and root biomass

On July 2, 2021, and July 5, 2022, five representative cotton plants were chosen to measure the individual morphological indices within each plot. The plant heights was measured using a straightedge.

#### 2.5.2 Measurement of the leaf canopy temperature and stomata

On July 4, 2021, and July 8, 2022, between 9:00 and 11:00 a.m., three representative plants were selected, and the third functional leaf was selected to measure the leaf canopy temperature and stomatal traits within each plot.

Canopy temperature was measured using a hand-held infrared thermometer (AGRI-THERM II, Model 6110, USA). The sensor probe was positioned 5 cm away from the upper third of the main stem functional leaf, which was perpendicular to the direction of leaf unfolding. Temperature measurements were recorded as soon as the probe was stable (Vantyghem et al., 2022).

Stomata were measured using a the nail polish and transparent tape technique (Baresch et al., 2019). Briefly, the nail polish was evenly applied evenly to the abaxial surface of the third functional leaf from the top and allowed to dry naturally for 2 to 5 min. The tape was then placed on the dried nail polish layer and attached to a slide without an additional coverslip. Images of the stomata were captured with an optical microscope (LEICA 2500, Wetzlar, Germany). Additionally, the length, width, opening and density of the stomata were determined on five randomly selected fields of view in each image (Wilson et al., 2009; Jiao et al., 2022).

#### 2.5.3 Measurement of the LRA

*Root sampling*: The LRA was determined on July 2, 2021, and July 5, 2022, using the “Shovelomics” method (Shao et al., 2019). This developmental stage is the critical period of cotton root system development when the root network is established. Three representative cotton plants were selected per plot. The soil volume around the plant root system (20 cm × 55 cm × 40 cm for the plant spacing × row spacing × depth, respectively) was excavated using a standard shovel. The root obtained was gently shaken to remove the adhering soil and then soaked in 0.5% detergent to remove the remaining soil particles. Finally, a low-pressure rinse was performed to remove any remaining soil particles and obtain a clean root system.

*Homemade imaging*: The imaging devices were composed of a laptop, an industrial digital camera (MER-500-14U3C; lens, M1224-MPW2 [Daheng Imaging, Beijing, China]), and an imaging tent. In its natural growth state, the root system was suspended from the top at the top of the tent and secured with spring clips. Subsequently, the Daheng Galaxy Viewer (x64) imaging software was utilized to adjust the optimal shooting parameters, including an exposure time of 65000 (us), a desired gray value of 120, and a white balance coefficient of 1.1484. Once the root system was properly fixed and stable, the images were captured without additional focus or light adjustments. The acquired root images were saved as JPEGs.

*Image processing with DeepLab V3C and RootNav*: The images were tagged and renamed for organization purposes. Next, the root images were segmented using DeepLab V3C (Zhao et al., 2022). Finally, the LRAs were extracted from the segmented root images using RootNav (Liao et al., 2001; Christopher et al., 2013).

#### 2.5.4 Measurement of the aboveground and root dry weight

The dry weight of the aboveground and root portions was determined using a weighing method, starting with the separation of the aboveground and root at the cotyledon node. Following this, the harvested leaf and root samples underwent heating at 105°C for 30 minutes and then at 80°C until a constant weight was achieved.

#### 2.5.5 Measurement of the yield and yield components

Cotton bolls were harvested from 20 plants in the center of each plot. The bolls were harvested on October 18, 2021, and October 15, 2022. The harvested cotton bolls were counted and weighed. Subsequently, the seed cotton from each harvest was collected, placed in nylon mesh bags, and stored in a dry room for 20 d for air drying, the seed cotton was weighed and then ginned. At the end of the drying period, the seed cotton in the plot was weighed (g) to calculate Weight per plant and seed cotton yield (kg hm^–2^, seed cotton yield =weight per plant (g) × harvest density/1000). Lint yield obtained by weighing after ginning.

### 2.6 Statistical analysis

Microsoft Excel 2019 (Redmond, WA, USA) was utilized for data entry. The quantitative data was analyzed by a one-way analysis of variance (ANOVA) using R version 4.1.2. Cluster analysis were drawn using the Origin 2019b Software Program (OriginLab, Northampton, MA, USA). The graphs were generated using GraphPad Prism 9.0 (GraphPad Software, Inc, San Diego, USA). Adobe Illustrator 2020 (Adobe, San Jose, CA, USA) was used to compose the images.

## 3 Results

### 3.1 The small and large LRAs

Varieties with small LRA and large LRA were selected using Euclidean distance flattening and systematic cluster analysis. In 2021, the varieties were clustered into five classes with a Euclidean distance of 0.5. Class I was composed of 25 (32%) varieties characterized by a small LRA. Class II consisted of 14 (18%) varieties characterized by a lesser LRA. Class III included 35 varieties, representing 44% of all the varieties, with an intermediate LRA. Class IV represented one variety, 1% of all the varieties, with a larger LRA. Category V was composed of four varieties, accounting for 5% of all the varieties, characterized by the largest LRA (**Supplementary Figure 4A**). In 2022, the varieties were clustered into four classes with a Euclidean distance of 0.5. Category I included 15 varieties, which represented 19% of all the varieties, with a small LRA. Category II was composed of 43 varieties (54% of all the varieties) characterized by a lesser LRA. Class III was composed of 20 varieties (26% of all the varieties) with a larger LRA. Category IV included one variety (1% of all the varieties) with the largest LRA (**Supplementary Figure 4B**).

Based on the two-year experimental results, three varieties with small LRAs were identified, variety #38, variety #77, and variety #7; and three varieties with large LRAs were identified, variety #48, variety #47, and variety #75 (**Supplementary Figure 4**). Further analysis will be conducted on these six varieties.

### 3.2 Phenotypic variation in the root-related traits

**Figure 1:**
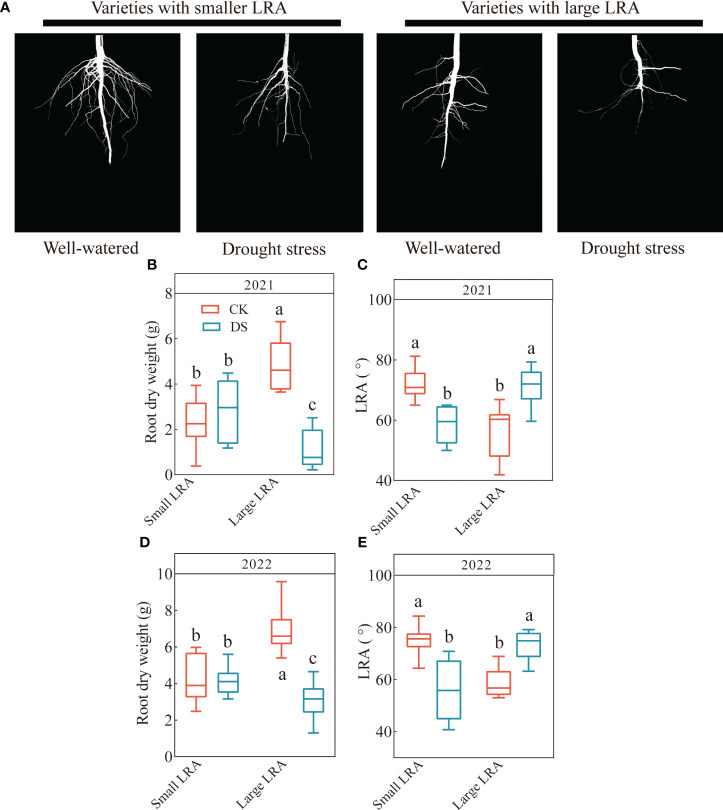
Responses of root traits to drought stress between three varieties with small LRAs and three varieties with large LRAs. Comparative charts illustrating different varieties based on LRA **(A)**; Quantitative analysis of the root dry weight **(B)** and LRA **(C)** in 2021; Quantitative analysis of the root dry weight **(D)** and LRA **(E)** in 2022. Different letters indicate significant differences in drought stress and between varieties at *p* < 0.05. WW, well-watered; DS, drought stress; LRA, lateral root angle. Values represent means ± standard error (n = 9). Red represents well-watered and blue represents drought stress.

There are genotypic differences among different LRA varieties under drought stress. Significant genotypic differences (*p* < 0.05) in the LRA were observed in both planting years (**Figure 1A**). In 2021, the range of the small LRA was 49.99 - 65.02 in varieties under drought stress and 59.65 - 79.33 for the large LRA. Drought stress significantly reduced the LRA in varieties with a small LRA and increased it in varieties with a large LRA compared to the well-watered (**Figure 1C**). Furthermore, under drought stress, the root dry weight and LRA were significantly reduced by 60% and increased by 22% in varieties with a large LRA compared to varieties with a small LRA (**Figures 1B, C**). In 2022, the range of the small LRA was 40.76 - 70.82 under drought stress and 63.16 - 79.17 for the large LRA (**Figure 1E**). Similarly, the root dry weight and LRA significantly increased by 26% and 32%, respectively, in the varieties with small LRAs under drought stress compared to the varieties with large LRAs (**Figures 1D, E**).

### 3.3 Phenotypic variation in the stomatal-related traits

**Figure 2:**
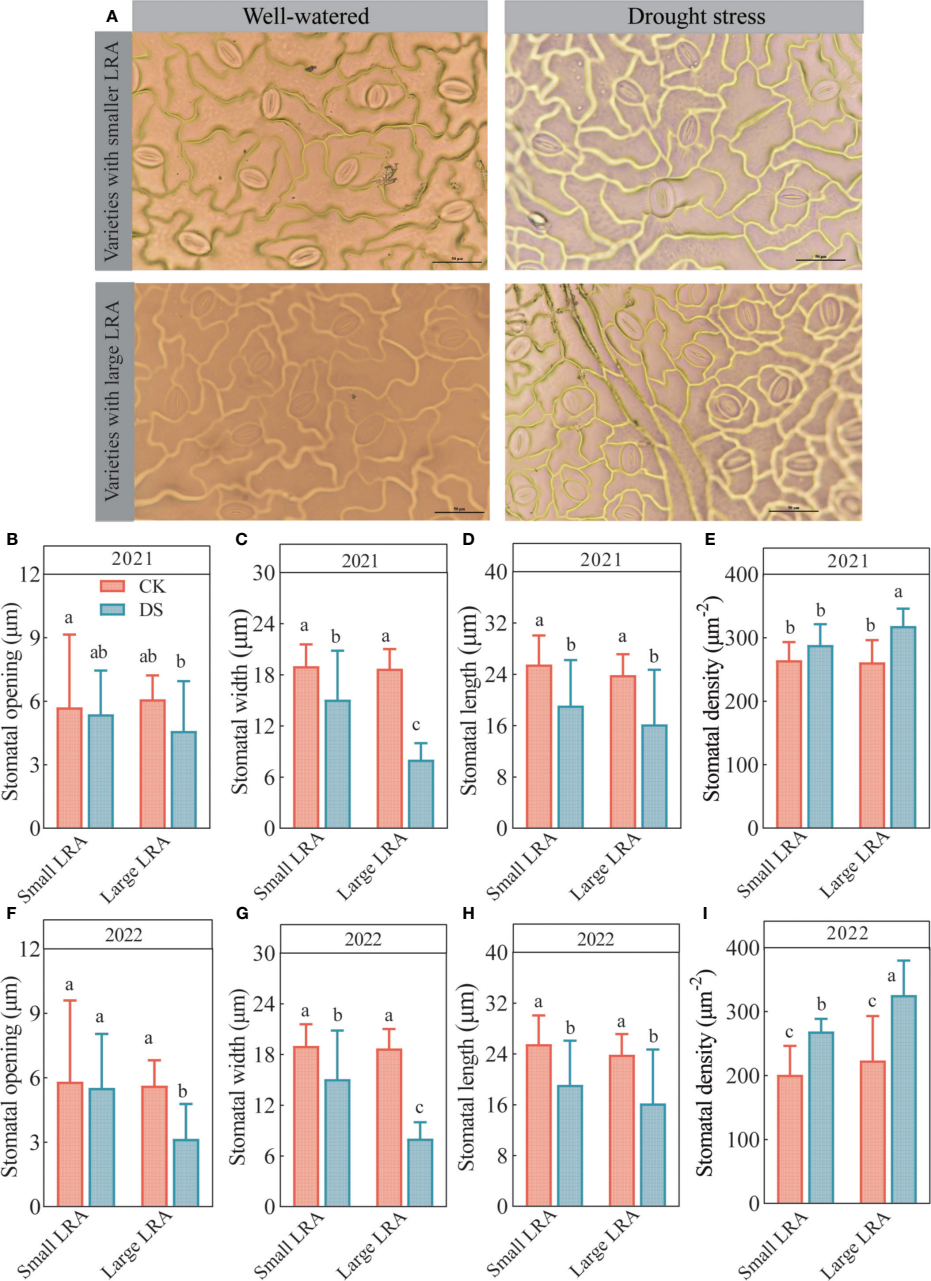
Responses of stomatal traits to drought stress between three varieties with small LRAs and three varieties with large LRAs. Comparative charts illustrating different varieties based on stomatal morphology **(A)**; Quantitative analysis of stomatal opening **(B)**, stomatal width **(C)**, stomatal length **(D)**, and stomatal density **(E)** in 2021; Quantitative analysis of stomatal opening **(F)**, stomatal width **(G)**, stomatal length **(H)**, and stomatal density **(I)** in 2022. WW, well-watered; DS, drought stress; LRA, lateral root angle. Different letters represent significant differences in drought stress and between varieties (*p* < 0.05). Values represent means ± standard error (n = 9). Red represents well-watered and blue represents drought stress.

Changes in the stomatal opening, length, width, and density in the leaves of cotton varieties with different LRAs under well-watered and drought stress are shown in **Figure 2A**. Under well-watered, the differences in the stomatal opening (**Figures 2B, F**), width (**Figures 2C, G**), length (**Figures 2D, H**), and density (**Figures 2E, I**) among the cotton varieties with the different LRAs were insignificant. However, the stomatal width, length, and opening were suppressed, while the stomatal density was promoted in varieties with large and small LRAs under drought stress.

Moreover, the cotton varieties with different LRAs demonstrated varying abilities to regulate stomatal opening under drought stress. Cotton varieties with small LRAs exhibited a stronger regulatory capacity, and varieties with large LRAs exhibited the weakest ability to regulate the stomatal openings (**Figures 2B, F**). Under drought stress in 2021, the stomatal density of the small LRA varieties was significantly reduced compared to the large varieties of LRA. In addition, the stomatal opening and width were significantly increased by 15% and 47% in the small LRA compared to the large LRA, respectively (**Figures 2B, C**). However, the differences in stomatal length were insignificant (**Figure 2D**). The stomatal density also reduced by 10% (**Figure 2E**). In 2022, under drought stress, varieties with small LRAs exhibited a significant increase of 43% in stomatal opening and 47% in stomatal length compared to varieties with large LRAs (**Figures 2F, H**), with no significant difference in stomatal width (**Figure 2G**). Additionally, the stomatal density was significantly reduced by 21% (**Figure 2**) for varieties with the small LRAs compared to those with the large LRAs.

### 3.4 Phenotypic variation in the leaf canopy temperature, leaf water potential and relative water content

**Figure 3:**
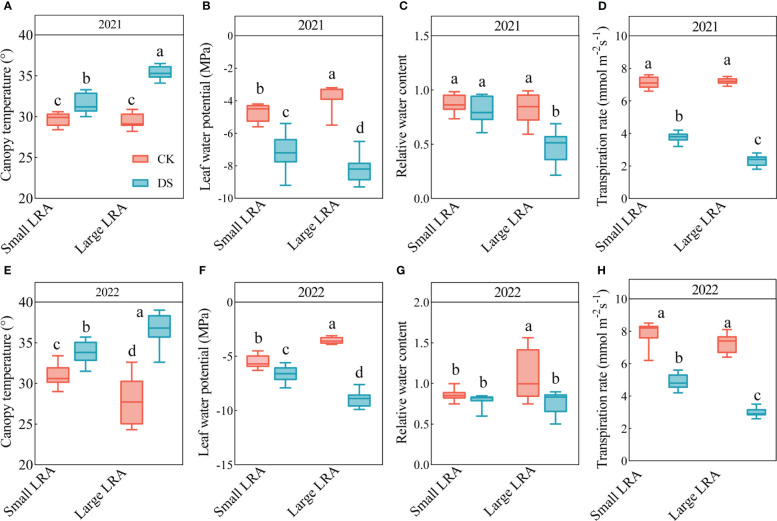
Responses of leaf physiological traits to drought stress between three varieties with small LRAs and three varieties with large LRAs. In 2021, we conducted statistical analyses of canopy temperature **(A)**, leaf water potential **(B)**, leaf relative water content **(C)**, and transpiration rate **(D)**. Similarly, in 2022, statistical analyses were carried out for canopy temperature **(E)**, leaf water potential **(F)**, leaf relative water content **(G)** and transpiration rate **(H)**. WW, well-watered; DS, drought stress; LRA, lateral root angle. Different letters indicate significant differences in drought stress and between varieties (*p* < 0.05). Values for canopy temperature in 2021 and 2022 are indicated by means ± standard errors (n = 9). Red represents well-watered and blue represents drought stress.

There were significant differences in the canopy temperature between 2021 and 2022 under drought stress (*p* < 0.05, **Figure 3**). In 2021, the canopy temperatures under drought stress were significantly higher than those under well-watered in plants with large and small LRAs (*p* < 0.05). However, the canopy temperature in varieties with small LRAs was significantly reduced by 12% under drought stress compared to varieties with large LRAs (**Figure 3A**). The same pattern persisted in 2022, although the canopy temperature in the varieties with small LRAs was reduced by 9% under drought stress compared to the varieties with large LRAs (**Figure 3E**).

Drought stress had an inhibitory effect on the leaf water potential for both the large and small LRAs (**Figures 3B, F**). Nevertheless, notable distinctions emerged in the response of LRAs to drought stress. In particular, in 2021 and 2022, the leaf water potential of the small LRAs exhibited a significant increase of 13.68% and 25.77%, respectively, compared to their counterparts with large LRAs under drought stress. The trend in relative water content of leaves with large and small LRAs under drought stress mirrored the patterns observed in the leaf water potential (**Figures 3C, G**).

Drought stress inhibited the transpiration rate of both large and small LRAs (**Figures 3D, H**). However, there were significant genotypic differences in the response of LRAs to drought stress. Under drought stress in 2021 and 2022, the transpiration rate of small LRAs varieties increased significantly by 61.24% and 62.00% compared to that of large LRAs varieties (**Figures 3D, H**).

### 3.5 Phenotypic variation in the plant height and aboveground biomass

Drought stress significantly impacted the plant height and aboveground dry matter mass across varieties with different LRAs (**Table 1**). In particular, drought stress reduced the height of plants with small LRAs by 36% (2021) and 6% (2022) compared to plants under well-watered treatment. Compared with the well-watered, drought stress reduced the height in the plants with large LRAs by 54% (2021) and 45% (2022). Additionally, the aboveground biomass of varieties with the small LRAs was reduced by 32% and 12% under drought stress in 2021 and 2022, respectively, compared to well-watered conditions. Alternatively, the aboveground biomass in the varieties with large LRAs was reduced by 75% and 71% in 2021 and 2022, respectively. Furthermore, under drought stress, the plant height and aboveground biomass in varieties with small LRAs increased by 23% and 54% in 2021 compared to the large LRAs. Similarly, in 2022, the plant height and aboveground biomass of varieties with small LRAs increased by 39% and 64%, respectively, compared to the large LRAs (**Table 1**).

**Table 1:** The plant height and aboveground biomass between varieties with small LRAs and large LRAs under well-watered and drought stress.

	Plant height (cm)				Aboveground biomass (g)			
	2021		2022		2021		2022	
	WW	DS	WW	DS	WW	DS	WW	DS
LRA group								
small LRA	36.19 ± 3.39	23.61 ± 2.24	56.22 ± 3.85	52.83 ± 3.33	25.08 ± 2.14	17.01 ± 6.29	41.13 ± 5.53	33.42 ± 7.80
large LRA	38.92 ± 4.01	17.83 ± 3.06	58.50 ± 5.44	32.16 ± 10.09	31.71 ± 7.05	7.90 ± 1.91	44.60 ± 6.77	14.44 ± 5.44
varieties								
#7	31.50 ± 1.22	25.67 ± 0.94	60.07 ± 1.80	55.10 ± 1.92	23.64 ± 3.73	16.29 ± 0.68	35.37 ± 0.85	28.66 ± 8.40
#77	37.67 ± 0.24	24.67 ± 0.47	50.97 ± 1.10	48.13 ± 0.97	23.50 ± 0.44	9.69 ± 3.95	39.43 ± 12.08	27.17 ± 0.77
#38	39.40 ± 4.17	20.50 ± 0.41	57.63 ± 1.23	55.27 ± 1.23	28.10 ± 2.06	25.04 ± 4.40	48.59 ± 4.36	44.42 ± 2.30
#47	34.43 ± 0.90	21.50 ± 0.00	57.33 ± 2.36	46.33 ± 1.25	22.04 ± 3.58	6.12 ± 0.21	51.05 ± 24.40	13.88 ± 0.80
#48	44.17 ± 2.25	18.00 ± 0.41	52.50 ± 1.08	23.67 ± 2.94	38.66 ± 1.21	10.55 ± 0.20	47.50 ± 13.78	21.36 ± 5.36
#75	38.17 ± 0.62	14.00 ± 0.41	65.67 ± 2.36	26.47 ± 4.23	34.42 ± 10.74	7.04 ± 1.39	35.25 ± 4.78	8.07 ± 3.31
Summary Statistics								
minimum	30.00	13.50	50.00	20.00	17.54	4.99	9.80	4.51
maximum	46.00	25.00	69.00	57.80	49.57	29.64	89.79	73.42
mean	37.56 ± 3.70	20.35 ± 2.65	57.36 ± 4.64	42.49 ± 6.71	28.39 ± 4.59	12.45 ± 4.10	42.86 ± 6.15	24.57 ± 6.62

LRA, lateral root angle; WW, well-watered; DS, drought stress; #7, #77, and #38 are varieties with small LRAs; #47, #48, and #75 are varieties with large LRAs.

### 3.6 Variation in seed cotton yield and related traits

**Figure 4:**
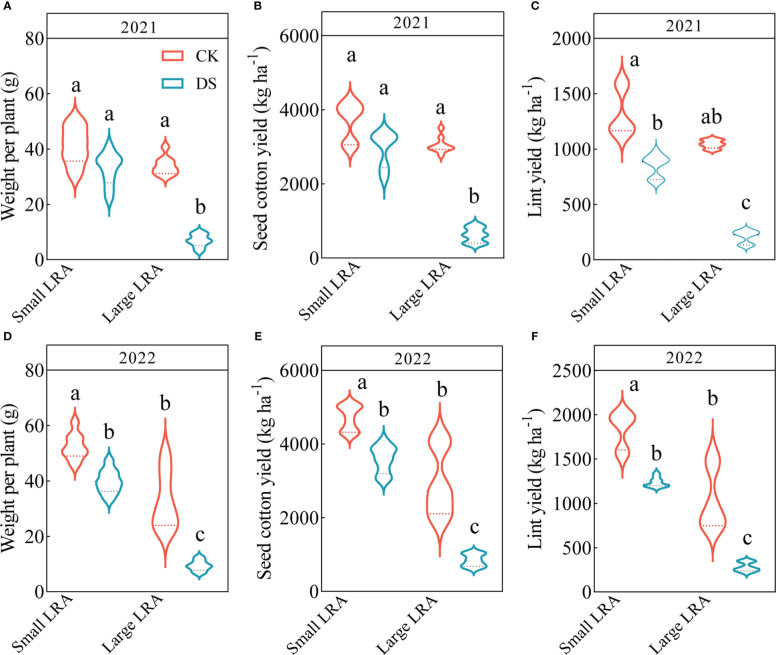
Responses of yield to drought stress between three varieties with small LRAs and three varieties with large LRAs. Quantitative analysis of the weight per plant **(A)**, seed cotton yield **(B)**, and lint yield **(C)** in 2021; Quantitative analysis of the weight per plant **(D)**, seed cotton yield **(E)**, and lint yield **(F)** in 2022. Different letters indicate significant differences in drought stress and between varieties (*p* < 0.05). WW, well-watered; DS, drought stress; LRA, lateral root angle. Values represent means ± standard error (n = 9). Red represents well-watered and blue represents drought stress.

Drought stress significantly reduced weight per plant, seed cotton yield and lint yield of cotton. Under drought stress in 2021, the seed cotton yields for varieties with small and large LRAs were 20% and 80% lower (**Figure 4B**); the lint yield was 36% and 80% lower (**Figure 4C**), and the weight per plant was 20% and 80% lower (**Figure 4A**), respectively, compared to under well-watered conditions. The same trend persisted in 2022, where the seed cotton yield was 24% and 71% lower (**Figure 4E**); the lint yield was 32% and 73% lower (**Figure 4F**), and the weight per plant was 24% and 71% lower (**Figure 4D**) under drought stress for varieties with small and large LRAs, respectively, compared to those under well-watered conditions.

There were genotypic differences in weight per plant, seed cotton yield and lint yield of cotton under drought stress. Under drought stress in 2021, the varieties with small LRAs recorded significantly increased seed cotton yield, lint yield and weight per plant by 79, 75 and 79%, respectively, compared to varieties with a large LRA (**Figures 4A-C**). Similarly, in 2022, varieties with smaller LRAs significantly increased seed cotton yield, lint yield, and weight per plant by 76%, 77%, and 76%, respectively, compared to varieties with larger LRAs (**Figures 4D-F**).

### 3.7 Relationships between the root system and key traits in field trials

**Figure 5:**
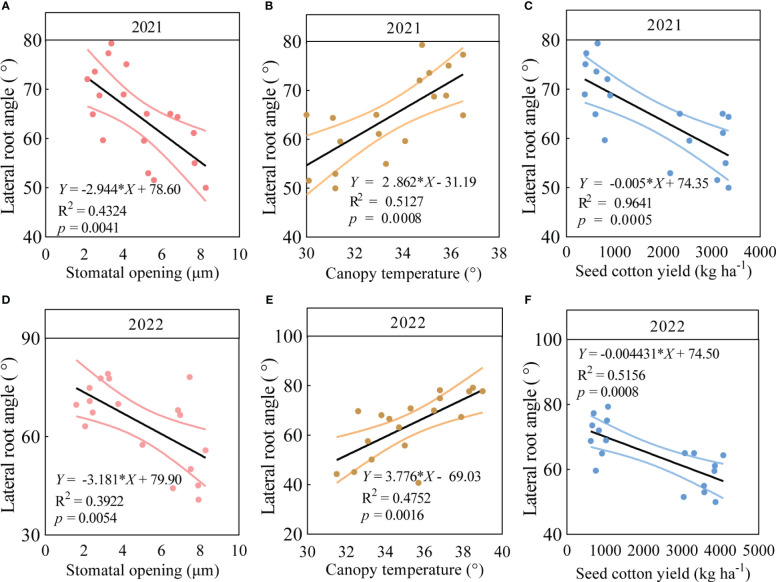
Correlation analysis between LRA and stomatal opening, canopy temperature, and seed cotton yield in three varieties with small LRAs and three varieties with large LRAs. In 2021, linear regression analysis was used to investigate the relationship between LRA and stomatal opening **(A)**, canopy temperature **(B)**, and seed cotton yield **(C)** in three varieties with small LRAs and three varieties with large LRAs under drought stress (n = 18); In 2022, linear regression analysis was used to investigate the relationship between LRA and stomatal opening **(D)**, canopy temperature **(E)**, and seed cotton yield **(F)** in three varieties with small LRAs and three varieties with large LRAs under drought stress (n = 18).

The LRA, stomatal opening and canopy temperature of the six varieties were significantly correlated with seed cotton yield under drought stress in 2021 and 2022. The findings revealed significant regressions (*p* < 0.05) between the LRA with stomatal opening, canopy temperature, and seed cotton yield in 2021 (**Figure 5**). In particular, in 2021, the LRA significantly increased with the increasing canopy temperature (*p* < 0.05, **Figure 5B**) and significantly decreased with the increasing stomatal opening (*p* < 0.05, **Figure 5A**) and seed cotton yield (*p* < 0.05, **Figure 5C**). Similarly, in 2022, the LRA significantly increased with the increasing canopy temperature (**Figure 5E**) and significantly decreased with the increasing stomatal opening (**Figure 5D**) and seed cotton yield (*p* < 0.05, **Figure 5F**).

## 4 Discussion

### 4.1 Cotton LRA under drought stress

Under drought stress, the root system’s capacity to delve into the soil becomes crucial because it enables the plants to extract water from deeper layers, which directly influences their growth (Adcock et al., 2007). Thus, measuring the root angle is vital to assess the drought tolerance of crops (Lynch, 2013; Lynch and Wojciechowski, 2015; Colombi and Walter, 2017).

Notably, previous research demonstrated that the root angle in barley (*Hordeum vulgare* L.) (Robinson et al., 2018) and rice (Uga et al., 2013) directly impacting their final yield. Moreover, MansChadi et al. (2010) further illustrated that with narrower roots, more soil water was extracted, especially at profile depth. Maize also provides an excellent example where the root angle proves to be a significant factor in increase crop yield under drought stress (Ali et al., 2015). MansChadi et al. (2008) revealed that drought-tolerant wheat genotypes had a narrower angle of seminal axes. Furthermore, the wheat varieties cultivated in arid regions tend to exhibit narrower root angles and a more compact root structure, which enhances their ability to access deep soil water (MansChadi et al., 2008). This study was consistent with the results of these findings. These findings are consistent with the results of this study. In the context of this study, different cotton varieties exhibited genetic differences in their LRAs. In particular, drought stress significantly reduced the LRAs of varieties with smaller LRAs and increased those of varieties with larger LRAs.

### 4.2 Stomata-related traits in cotton under drought stress

This study explores the complex relationship between cotton LRA and stomatal traits under drought stress. Stomata are a vital component of the regulation of water by the plant under drought stress conditions. Generally, the leaf stomatal density increases under mild to moderate stress levels or at the onset of stress, such as drought stress (Fraser et al., 2009). This study was consistent with Fraser et al. (2009). We observed an increase in stomatal density in varieties with small and large LRAs under drought stress compared to well-watered conditions.

The extent of the increase in stomatal density varies among types of crops. In this study, we found that under drought stress, varieties with smaller LRAs had a 10% and 21% reduction in stomatal density compared to those with larger LRAs in 2021 and 2022, respectively. Additionally, we observed that under drought stress, as the stomatal density increased, the stomatal width, length, and aperture decreased. This is consistent with the results of Hetherington et al. (2003), who indicated that the stomatal size decreases with increasing stomatal density. Plants exchange gases, including water vapor, through stomata. This is particularly important for plants grown under limited water resources (Gray and Hetherington, 2004; Shimazaki et al., 2008; Yoo et al., 2009; Zhang et al., 2018). Drought stress reduced the stomatal opening in metatarsal leaves (Grantz and Schwartz, 1988), soybean (Ohashi et al., 2006), and winter wheat (Li et al., 2004), which is consistent with the results of this study. Our research showed that the stomata of varieties with large and small LRAs reduced their degree of stomatal opening under drought stress. However, the extent of the reduction of stomatal opening varied among crop types. We found that compared to cotton varieties with larger LRAs, those with smaller LRAs increased their stomatal opening by 15% and 43% under drought stress in 2021 and 2022, respectively. Our results are in agreement with Vinarao et al. (2023) who showed that the narrow root cone angle rice genotypes have higher stomatal conductance compared to the wide root cone angle rice genotypes.

Overall, drought-tolerant varieties can more easily access deep water, and the amount of water accessed can be greater to compensate for the loss of large transpiration caused by stomatal opening, which can improve the drought tolerance of plants, leading to better survival and growth of plants under drought stress conditions.

### 4.3 Canopy temperature in cotton under drought stress

The canopy temperature is affected by genetic, environmental, and field management factors (Balota et al., 2007; Reynolds et al., 2009). The exchange of energy between the canopy and the external environment happens through radiation, conduction, convection, transpiration, and various metabolic processes, including enzymatic reactions, respiration, and photosynthesis (Yoshimoto et al., 2005). In various crops, such as upland rice, sugar beet (*Beta vulgaris* subsp. *vulgaris* Altissima Group), and potato (*Solanum tuberosum* L.), the difference between the canopy and air temperatures indicate drought tolerance (Fukuoka et al., 2003). Therefore, the canopy temperature is a crucial indicator to monitor the responses of plants to drought stress.

This study delved into the effects of drought stress on cotton canopy temperature, and it is closely related to the LRA. During drought stress, elevated canopy temperatures may increase the rates of plant transpiration and accelerate the evaporation of soil water, which increases the plant’s water needs (Carvalho et al., 2020). However, excessively high canopy temperatures can trigger heat stress responses in plants, which disrupts their normal growth and development (Mohammed et al., 2018). Moller et al. (2006) also suggested a model to estimate the water status of grape (*Vitis vinifera* L.) using the canopy temperature. Different plant varieties exhibit varying adaptations to temperature. This study shows that, under drought stress conditions, cotton plants experienced a notable increase in canopy temperature. However, in the years 2021 and 2022, cotton varieties with smaller LRAs exhibited a significant reduction in the canopy temperature by 12% and 9%, respectively, when compared to varieties with larger LRAs under drought stress. This results is consistent with Vinarao et al. (2023), who showed that compared to rice genotypes with wide root cone angle, the narrow root cone angle group had a 0.3-1.0° C decrease in canopy temperature below 20 centimeters.

The reduction in canopy temperature among in cotton varieties with smaller LRAs suggests an improved ability to regulate water use under drought stress. This adaptation enables these plants to adapt to limited water availability more effectively, which ultimately results in improved drought tolerance and potentially higher yields. These findings underscore the intricate relationship between the root system traits, canopy temperature, and drought tolerance in cotton.

### 4.4 The relationship between the LRA, canopy temperature, stomatal traits, and seed cotton yield

Our research unveiled a compelling correlation between the LRA and leaf stomatal opening, canopy temperature, and yield. Research has shown that cotton varieties with larger LRAs may lead to elevated leaf temperatures during drought stress. Conversely, under drought stress conditions, cotton varieties with smaller LRAs consistently displayed lower leaf canopy temperatures. This phenomenon arises from their enhanced ability to efficiently access water deep within the soil, which potentially induces greater transpiration in plants, thus, ameliorating water stress. These findings align closely with the research by Kato and Okami (2010) in which deeper-rooted varieties maintained open stomata and sustained lower leaf temperatures even when soil water potential dropped below -50 kPa, which illustrated the varietal adaptation of stomatal traits to drought stress. This discovery emphatically underscores the pivotal role played by the LRAs in facilitating the access of plants to deeper soil layers, thereby alleviating water deficits in the topsoil. The resultant reduction in leaf canopy temperatures suggests the potential of smaller LRAs to mitigate the heat stress induced by drought.

Furthermore, since the plant leaf temperature is typically influenced by the canopy temperature and controlled by the stomatal opening, our study highlights the significant correlation between the LRA and stomatal traits. The LRA affects the internal water transport in plants by influencing the stomata opening. We found that under drought stress, cotton varieties with smaller LRAs had relatively increased stomatal openings compared to varieties with larger LRAs. This could be because cotton plants with smaller LRAs can typically access deeper soil water, maintain a higher water supply, alleviate drought stress, and enable sufficient stomatal openings to support transpiration losses. In contrast, cotton plants with larger LRAs may face a lower water supply and be unable to sustain transpiration losses. Thus, they respond to drought stress by closing their stomata. In particular, under drought stress, cotton varieties with smaller LRAs exhibited a 15%-43% increase in their stomatal opening compared to varieties with larger LRAs. These results are consistent with those of Kato and Okami (2010) and Vinarao et al. (2023), who demonstrated that, varieties with deeper or narrower root systems maintained larger stomatal openings and higher stomatal conductance under drought stress. Therefore, this response is crucial for water resource conservation since it is sufficient to maintain a higher water supply, alleviate drought stress, and improve the plant’s resistance to water deficits.

Importantly, these findings significantly impact seed cotton yield. Under drought stress conditions, cotton varieties with smaller LRAs consistently outperform other varieties, primarily owing to their ability to access more water resources, thereby increasing seed cotton yield. However, cotton varieties with larger LRAs may be subject to water limitations, which results in reduced yields. Our research indicates that the seed cotton yield, lint yield, and individual plant weight of varieties with smaller LRAs increased significantly by 76%-79%, 75%-77%, and 76%-79%, respectively, compared to varieties with larger LRAs. These results are consistent with those of MansChadi et al. (2010), who demonstrated that wheat genotypes with narrow root angles exhibit 6%-28% higher yields than conventional varieties.

This correlation between the LRA and canopy temperature, stomatal opening, and yield strongly suggests the potential to use the LRA as a key trait in breeding programs aimed at developing drought-resistant cotton varieties. Recent studies provide support for such interrelationships. Vinarao et al. (2023) published research that illustrated a similar relationship, albeit in a different crop context. In particular, the RCA had significant negative correlations with the total root length (r = -0.70), leaf area (r = -0.67), proportion of deep roots below 20 cm (r = -0.65), and stomatal conductance (r = -0.71). Conversely, it had a significant positive correlation with canopy temperature (r = 0.66). These findings align closely with our results where we identified a negative correlation between the LRA and stomatal opening and seed cotton yield along with a positive correlation with canopy temperature.

In summary, the reduction in stomatal aperture reduces water loss, but also weakens photosynthesis and transpiration, and blocks assimilate transfer and heat dissipation in the plant body, a large increase in temperature will cause irreversible damage to plant organs and the yield loss of cotton will be greater (Sánchez et al., 2014). This understanding provides valuable insights into the mechanisms by which cotton adapts to water scarcity, thus, offering a promising avenue for precision breeding efforts. By selecting cotton varieties with smaller LRAs, breeders can facilitate the development of more robust cotton crops, ensuring sustainable production in water-limited environments and addressing the challenges posed by evolving climate patterns.

### 4.5 More needs to be done

The evaluation of drought tolerance using physiological mechanisms has made little progress. There are three main issues at the heart of this dilemma. First, the specific plant traits that enhance drought tolerance remain uncertain. Secondly, whether plant traits represent mutually exclusive substitutes, trade-offs, or orthogonal processes is unclear. Third, it is unclear whether some traits should be included in the drought stress treatment group. This study has addressed the first and second challenges. Our results demonstrate that the LRA, leaf canopy temperature, and stomatal opening are important factors in improving plant water relations and achieving higher seed cotton yield under drought stress. Despite the progress in linking LRA phenotypes and performance, the genetic basis of LRA remains poorly understood and merits further investigation.

## 5 Conclusion

**Figure 6:**
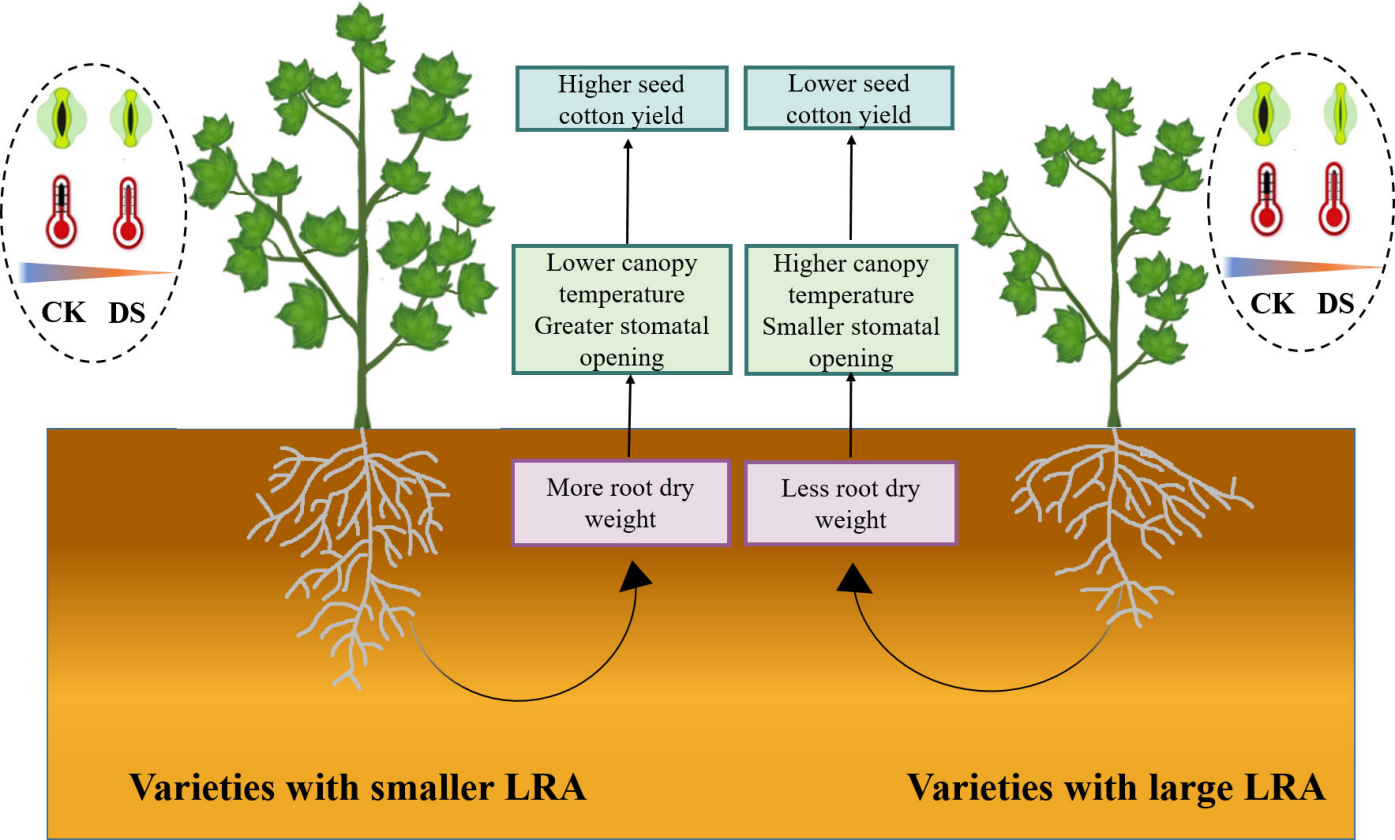
Physiological mechanisms of different LRA in response to plant drought stress. This study delves into the comparative analysis of drought tolerance regulatory mechanisms in cotton varieties featuring varying LRAs under drought stress. In the cotton varieties with a smaller LRA, a higher root dry weight is observed, contributing to enhanced aboveground drought tolerance. This enhancement is evidenced by a lower canopy temperature and a relatively suitable stomatal aperture under drought stress, ultimately mitigating the loss of seed cotton yield. However, the regulatory mechanism of LRA exhibits variations among plant species and fluctuates concerning drought severity and specific growth stages. Notably, cotton varieties with a large LRA demonstrate comparatively lower drought tolerance when contrasted with their smaller LRA counterparts. WW, well-watered; DS, drought stress; LRA, lateral root angle.

This study offers a comprehensive insight into the intricate connections between the cotton LRA, stomatal traits, canopy temperature, and yield under drought stress. Our study emphasizes the pivotal significance of the LRA as a hereditary trait that profoundly impacts how cotton responds to water scarcity. This study reveals that in drought stress, cotton varieties with smaller LRAs positively influence both yield and plant adaptability. Smaller LRAs could more easily access deep water, allowing the plants to obtain more water to compensate for the massive transpiration water loss caused by stomatal opening, thus, reducing the degree of plant water stress. As a result, smaller LRAs are better able to maintain stomatal opening. Additionally, cotton plants with smaller LRAs display improved stomatal regulation, which leads to lower canopy temperatures. Smaller LRAs are linked to reduced canopy temperatures. In summary, smaller LRAs significantly influence the aboveground growth of cotton, enhances drought tolerance, and reduces yield losses. Cotton varieties with smaller LRAs exhibit higher drought tolerance and seed cotton yields, primarily owing to their capacity to access deep soil water, thereby increasing plant water acquisition, lowering canopy temperatures, and permitting partial stomatal opening (**Figure 6**). Consequently, the LRA of cotton varieties proves critical in the selection of genotypes adapted to drought stress, and high yield potential.

## Data availability statement

The original contributions presented in the study are included in the article/**Supplementary Material**, further inquiries can be directed to the corresponding author/s.

## Author contributions

CG: Conceptualization, Methodology, Data curation, Formal analysis, Visualization, Writing – original draft. XB: Conceptualization, Data curation, Formal analysis, Methodology, Visualization, Writing – original draft, Validation. HS: Conceptualization, Methodology, Writing – original draft, Software. JC: Conceptualization, Methodology, Software, Writing – original draft. LZ: Conceptualization, Methodology, Software, Writing – original draft. JZ: Data curation, Formal analysis, Supervision, Writing – review & editing. HZ: Data curation, Formal analysis, Supervision, Writing – review & editing. YZ: Writing – review & editing, Visualization. KZ: Visualization, Writing – review & editing. ZB: Visualization, Writing – review & editing. AL: Methodology, Writing – original draft. LL: Methodology, Conceptualization, Project administration, Supervision, Writing – review & editing. CL: Conceptualization, Methodology, Project administration, Supervision, Writing – review & editing.

## Supplementary material

Supplementary Table 1 Detailed information on the 80 cotton varieties.

Supplementary Table 2 The soil bulk density and field water capacity at the 0-60 cm soil layer.

Supplementary Figure 1 The mean temperature and sunshine duration during the cotton growing seasons in 2021 **(A)** and 2022 **(B)**.

Supplementary Figure 2 The soil relative water content in 2021 **(A)** and 2022 **(B)** in the experimental fields. The data were recorded from April 24 to October 15 (n = 4021). WW, well-watered; DS, drought stress.

Supplementary Figure 3 Soil relative water content in cotton fields. The relative water content of the soil was 75 ± 5% in 2021 **(A)** and 2022 **(B)**. The relative water content of the soil was 50 ± 5% in 2021 **(C)** and 2022 **(D)**.

Supplementary Figure 4 Systematic clustering based on LRA drought tolerance coefficients for 80 cotton varieties in 2021 **(A)** and 2022 **(B)**.
