# Supplementary material for: The crucial role of lateral root angle in enhancing drought resilience in cotton
Source: Front Plant Sci. 2024 Feb 5;15:1358163. doi: 10.3389/fpls.2024.1358163 (PMC10875062; doi:10.3389/fpls.2024.1358163)
Supplement: Supplementary Table 1 — Detailed information on the 80 cotton varieties. [file DataSheet_1.docx]

**SUPPLEMENTARY TABLE 1** Detailed information on the 80 cotton varieties

| **No.** | **Variety** | **Authorized number** | **No.** | **Variety** | **Authorized number** |
| --- | --- | --- | --- | --- | --- |
| 1 | Jifeng 554 | Jishenmian 2009003 | 41 | Hanwu 216 | Jishenmian 2014010 |
| 2 | Jifeng 103 | Jishenmian 20190008 | 42 | Zhongmian 100 | Guoshenmian 2016003 |
| 3 | Jifeng 522 | Jishenmian 20050552 | 43 | Zhongmiansuo 79 | Yushenmian 2010006 |
| 4 | Jifeng 908 |  | 44 | Cangmian 666 | Lushenmian 20160030 |
| 5 | Jifeng 914 | Guoshenmian 2015003 | 45 | Han 6203 | Guoshenmian 2015002 |
| 6 | Jifeng 1982 | Jishenmian 2014001 | 46 | Shikang 126 | Guoshenmian 2008002 |
| 7 | Jifeng 4 | Guoshenmian 20210020 | 47 | Cang 198 | Jishenmian 2007006 |
| 8 | 7886 | GSM08003 | 48 | Ji 228 | Guoshenmian 2008003 |
| 9 | Cangmian 268 | Lushenmian 20160030 | 49 | Guoxinmian 9 | Guoshenmian 2009004 |
| 10 | Jimian 315 | Jishenmian 20190010 | 50 | K836 | Lushenmian 2012018 |
| 11 | Han 218 | Jishenmian 2015003 | 51 | Lumian 522 | Lushenmian 20170041 |
| 12 | Hannong 12 |  | 52 | Lumian 5172 |  |
| 13 | Han 8266 | Guoshenmian 2014001 | 53 | K638 | Lushenmian 2010010 |
| 14 | Han 258 | Jishenmian 2015009 | 54 | Guoxin 4 | Jishenmian 2006008 |
| 15 | Han 686 | Yushenmian 2011010 | 55 | Jifeng1187 | Jishenmian 20200005 |
| 16 | YM111 | Guoshenmian 20170002 | 56 | Jifeng 1458 | Jishenmian 20200004 |
| 17 | Nongda KZ05 | Jishenmian 2013006 | 57 | Jifeng 103 | Guoshenmian 20190014 |
| 18 | Nongdamian 10 | Jishenmian 2015007 | 58 | Jifeng 914 | Guoshenmian 2015003 |
| 19 | Nongdamian 12 | Jishenmian 2014011 | 59 | Jifeng 965 |  |
| 20 | Lumianyan 28 | Guoshenmian 2006012 | 60 | MH335223 | Jishenmian 20190018 |
| 21 | Xuzhou 1818 | I3A01080 | 61 | Guoxinmian 11 | Guoshenmian 2009001 |
| 22 | Zhongmiansuo 41 | Guoshenmian 2002001 | 62 | Zhongmiansuo 17 | xinshemmian19980071991 |
| 23 | Shandongxiamian11-42 |  | 63 | Chunbeibao |  |
| 24 | Zhongmiansuo 12 | Lushenmian 0064 | 64 | Zhongmiansuo 60 | Zheshenmian 2007002 |
| 25 | Yumian 19 | Guoshenmian 2001002 | 65 | CG3020-3 |  |
| 26 | Ejing 1 | GS08002-1991 | 66 | Jimian 2016 | Jishenmian 20199002 |
| 27 | Zhongmiansuo 35 | Guoshenmian 990005 | 67 | Ji 1518 | Jishenmian 2014005 |
| 28 | Zhongmiansuo 60 | Shanshenmian 2013001 | 68 | Jihang 8 | Jishenmian 20190009 |
| 29 | Xinshi 71143 | Guoshenmian 2014004 | 69 | Jimian 262 | Jishenmian 20200002 |
| 30 | Xinza 15 | Jishenmian 2014007 | 70 | Ji 178 | Jishenmian 2015010 |
| 31 | Xinshi 17 | Jishenmian 2015004 | 71 | Ji 172 | Jishenmian 20210003 |
| 32 | GK39 | Guoshenmian 2015008 | 72 | Yuzaomian 9110 | Yushenmian 2012006 |
| 33 | 0 shi |  | 73 | Dexiamian 1 | Lushenmian 0220 |
| 34 | Zhongmiansuo 94A915 | Jinshenmian 2016002 | 74 | Jicai 6913 |  |
| 35 | Lumianyan 36 | Lushenmian 2009022 | 75 | Zhongmiansuo 23 | Guoshenmian 980007 |
| 36 | DP33B |  | 76 | Zhongmiansuo 50 | Guoshenmian 2007013 |
| 37 | Guoxinmian01 | Yushenmian 2009004 | 77 | Ji668 | Guoshenmian 2001001 |
| 38 | Guoxinmian02 |  | 78 | Zhibao 86-1 | GS08009-1984 |
| 39 | Guoxinmian03 | Guoshenmian 2006003 | 79 | Jimian 958 | Guoshenmian 2006005 |
| 40 | Guoxinmian05 | Guoshenmian 2006003 | 80 | Jifeng 1271 | Jishenmian 2012002 |

**SUPPLEMENTARY TABLE 2** The soil bulk density and field water capacity at the 0-60 cm soil layer.

| **Soli layer (cm)** | **Bulk density (g cm^-3^)** | **Field capacity (%)** |
| --- | --- | --- |
| 0-20 | 1.33 | 34.9 |
| 20-40 | 1.52 | 35.2 |
| 40-60 | 1.53 | 35.3 |

**
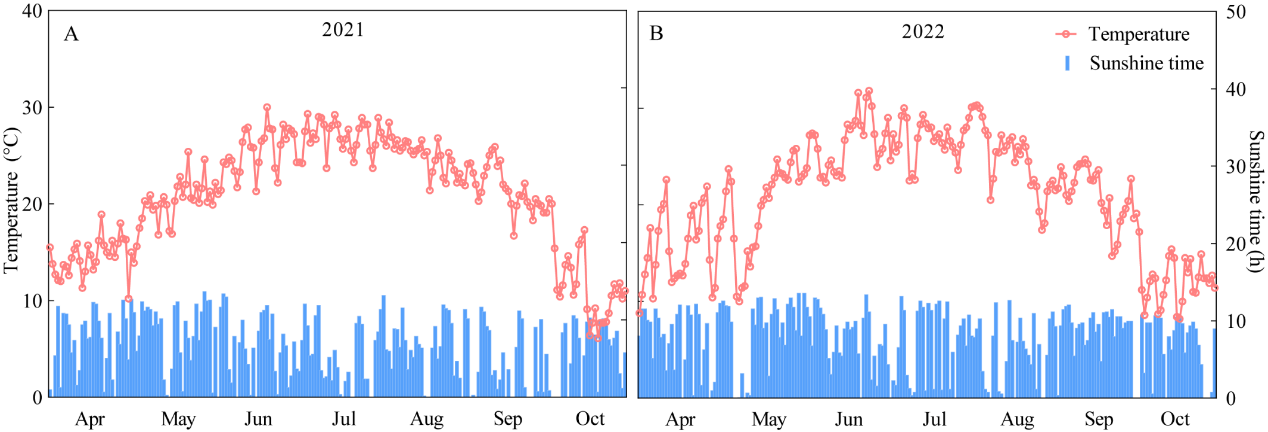
**

**SUPPLEMENTARY FIGURE 1** The mean temperature and sunshine duration during the cotton growing seasons in 2021 (A) and 2022 (B).


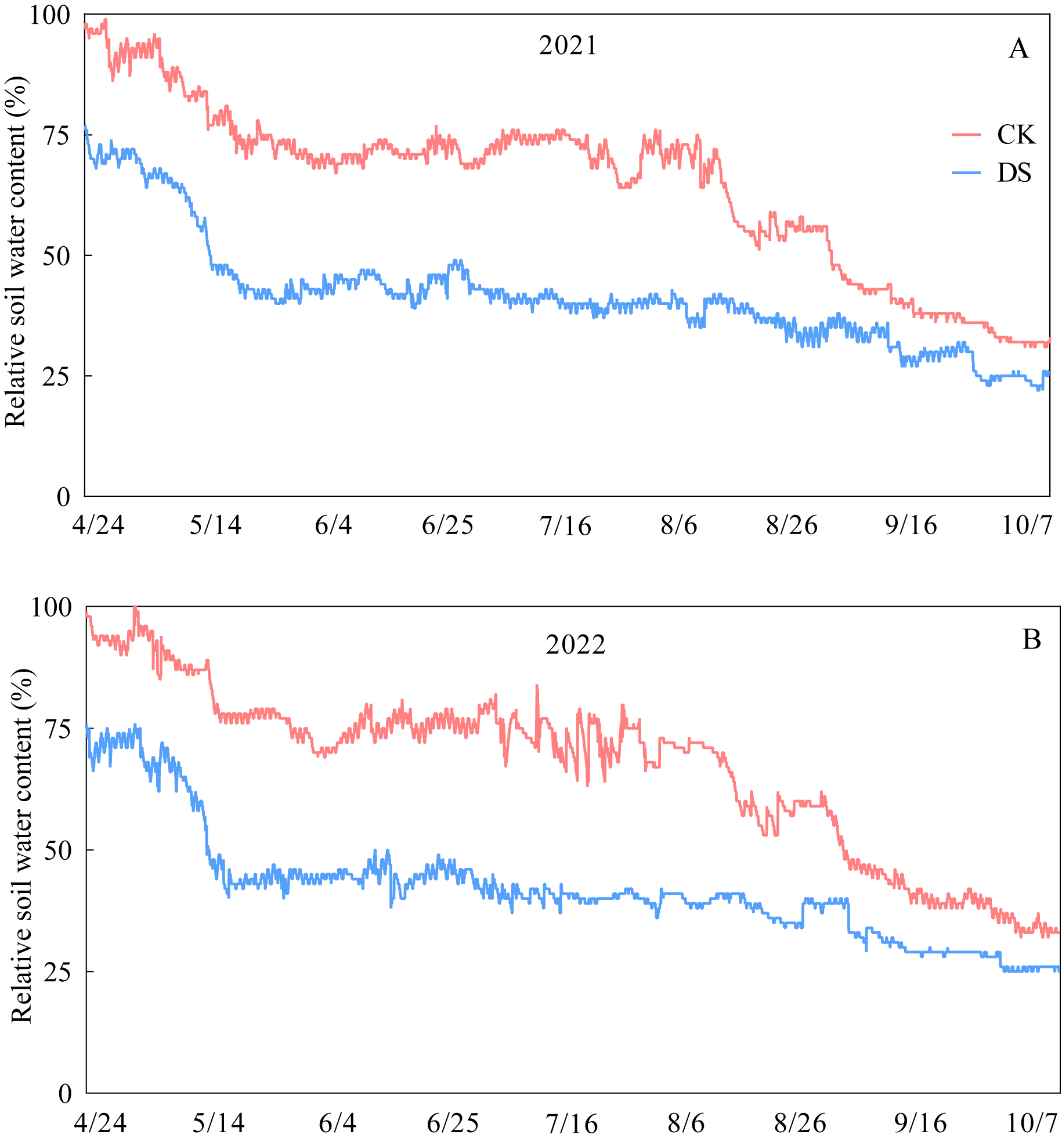


**SUPPLEMENTARY FIGURE 2** The soil relative water content in 2021 (A) and 2022 (B) in the experimental fields. The data were recorded from April 24 to October 15 (n = 4021). WW, well-watered; DS, drought stress.


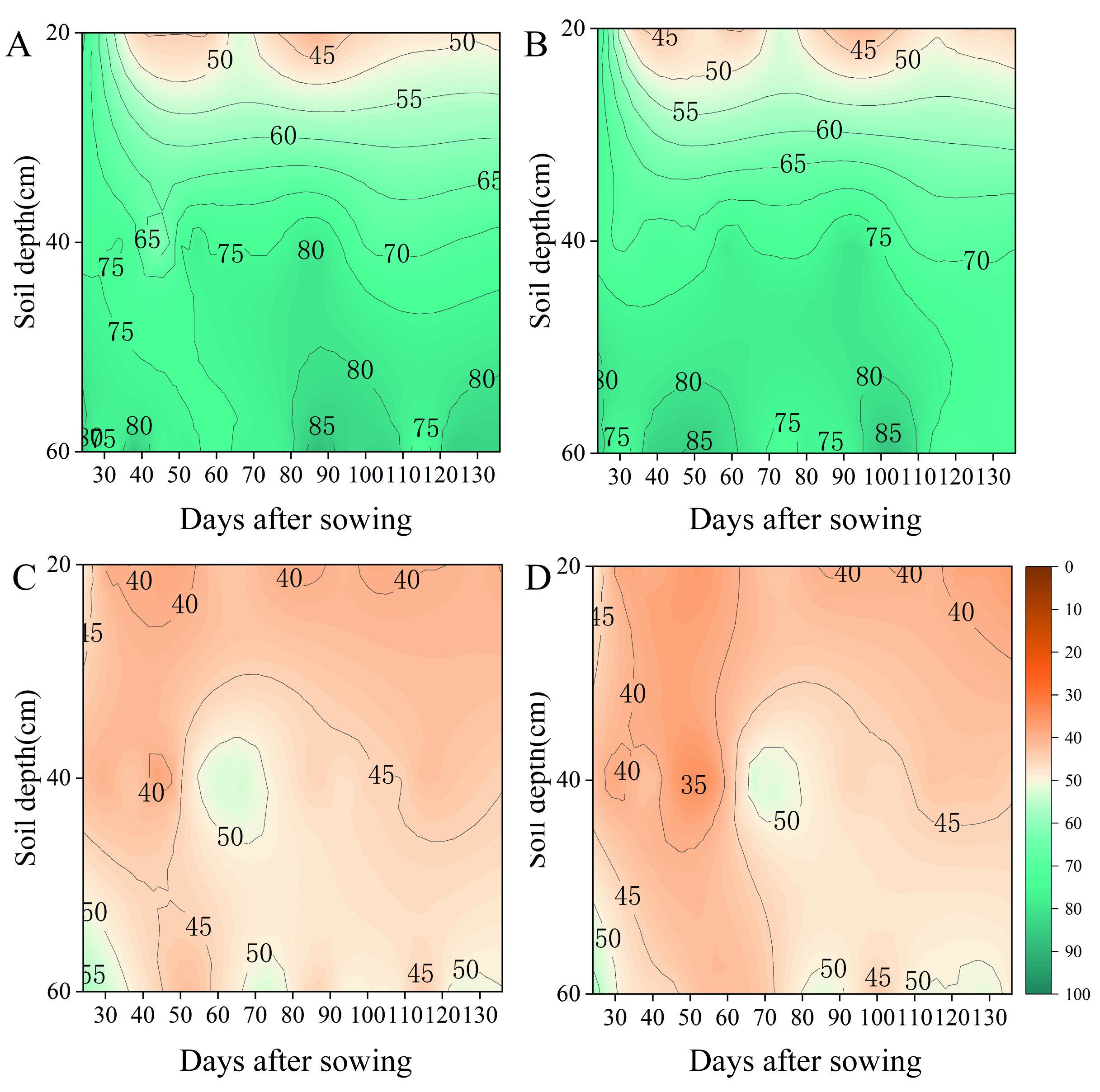


**SUPPLEMENTARY FIGURE 3** Soil relative water content in cotton fields. The relative water content of the soil was 75±5% in 2021 (A) and 2022 (B). The relative water content of the soil was 50±5% in 2021 (C) and 2022 (D).


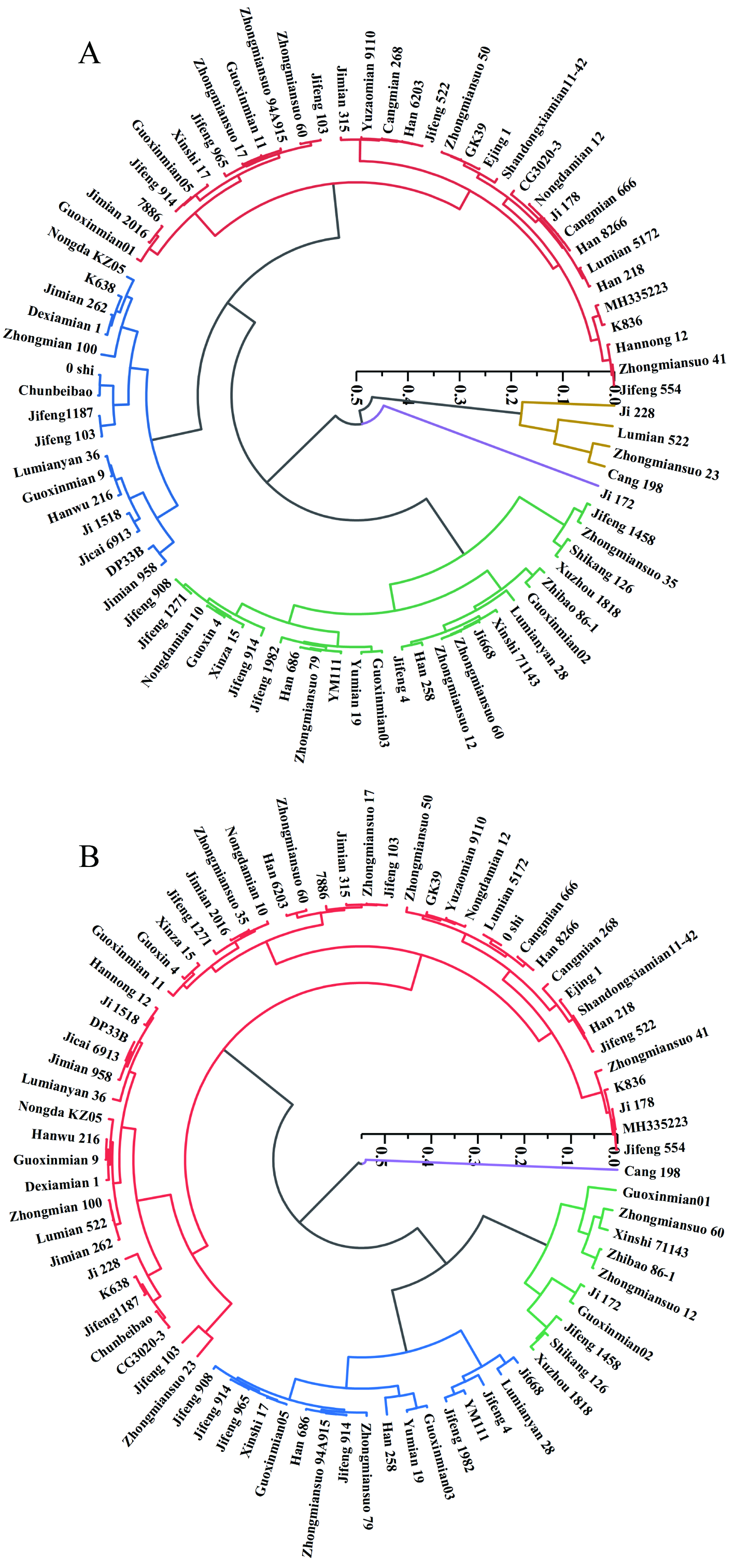


**SUPPLEMENTARY FIGURE 4** Systematic clustering based on LRA drought tolerance coefficients for 80 cotton varieties in 2021 (A) and 2022 (B).
